# Correlations between Capsular Changes and ROM Restriction in Frozen Shoulder Evaluated by Plain MRI and MR Arthrography

**DOI:** 10.2174/1874325001812010396

**Published:** 2018-10-17

**Authors:** Kenji Kanazawa, Yoshihiro Hagiwara, Takuya Sekiguchi, Kazuaki Suzuki, Masashi Koide, Akira Ando, Yutaka Yabe

**Affiliations:** 1 Department of Orthopaedic Surgery, South Miyagi Medical Center, Oogawara, Miyagi, Japan; 2 Department of Orthopaedic Surgery, Tohoku University School of Medicine, Sendai, Miyagi, Japan; 3 Department of Orthopaedic Surgery, Iwate Prefectural Central Hospital, Morioka, Iwate, Japan; 4 Department of Orthopaedic Surgery, JR Sendai Hospital, Sendai, Miyagi, Japan; 5 Department of Orthopaedic Surgery, Matsuda Hospital, Sendai, Miyagi, Japan

**Keywords:** Range of motion, Restriction, Coracohumeral ligament, Thickness, Magnetic resonance imaging, Magnetic resonance arthrography

## Abstract

**Background:**

Evaluation of the Range Of Motion (ROM) is one of the important procedures for shoulder disorders. The purpose of this study was to investigate correlations between capsular changes and ROM restrictions evaluated by both plain magnetic resonance imaging (MRI) and Magnetic Resonance Arthrography (MRA) in the same patients with frozen shoulder.

**Methods:**

Between March 2015 and June 2016, 24 patients with frozen shoulders (13 male and 11 female patients, mean age 60.5) with severe ROM restriction who underwent both MRI and MRA on the same affected side were evaluated. We evaluated 1) ROM, 2) the coracohumeral ligament (CHL) thickness, 3) the joint capsule thickness in the axillary recess (humeral and glenoid sides), 4) the area of the axillary recess, and 5) the capsular area of the axillary recess.

**Results:**

Positive correlations were found between the axillary area and forward flexion (FF) (R = 0.43, P = 0.035), lateral elevation (LE) (R = 0.66, P<0.001), external rotation (ER)(R = 0.43, P = 0.035), 90° abduction with external rotation (AER)(R = 0.56, P = 0.004), and hand behind the back (HBB)(R = 0.6, P = 0.002) on MRA. Negative correlations were found between the joint capsule at the glenoid side and ER and HBB in both MRI and MRA.

**Conclusion:**

The axillary area was significantly correlated with ROM restriction in FF, LE, ER, AER, and HBB on MRA. Thickness of the joint capsule at the glenoid side is an important factor for ROM restriction in frozen shoulder.

**Level of Evidence:**

Level 3, Study of Diagnostic Test.

## 1 INTRODUCTION

Evaluating the Range Of Motion (ROM) is an important step for diagnosis and deciding therapeutic approaches for patients with shoulder disorders. Frozen shoulder is a common shoulder disease characterized by both active and passive ROM restriction with severe pain [1]. Its pathogenesis has been unclear and there has been no international standard definition for it. Additionally, because of the ambiguity of the frozen shoulder definition, the degree of ROM restriction and its way of measurement (including scapulohumeral or only the glenohumeral motion) are often different according to researchers [2-4], which makes it difficult to compare results.

Plain Magnetic Resonance Imaging (MRI) as well as Magnetic Resonance Arthrography (MRA), intra-articular injection of a gadolinium-based contrast agent, is a useful diagnostic method for evaluating shoulder disorders [5, 6]. MRA can clearly visualize the rotator interval (RI), containing the coracohumeal ligament (CHL), by expanding the joint capsule [7, 8]. Characteristics of frozen shoulder by MRA are obliteration of the inferior axillary recess and a small capsular volume [9]. Numerous studies of MRI and MRA for frozen shoulder focused on the thickening of the CHL and obliteration of the fat triangle below the coracoid process [5, 6, 10, 11]. The CHL envelops the entire subscapularis (SSC) as well as its insertion, and it appears to function as support for the SSC and supraspinatus (SSP) muscles [12]. Its thickness is directly correlated to restriction in external rotation with the arm at side (ER) [13, 14] as well as in internal rotation (IR) by MRA [5]. Furthermore, for patients with anterior glenoid humeral instability, obliteration of the subcoracoid fat triangle and the CHL thickness evaluated by MRA correlated with ROM restrictions, such as Forward Flexion (FF), ER, and Hand Behind the Back (HBB) [15]. The CHL assumes to influence multiple ROM restrictions in shoulder disorders.

Besides the CHL and RI, thickening of the joint capsule and reduction of volume at the axillary recess strongly correlated with limited ROM or pain in patients with frozen shoulder evaluated by MRI [16, 17]. Some reports showed that thickening of the capsule in the axillary recess was a significant sign of frozen shoulder [16, 18], but others were not by MRA [5, 19]. As for the volume of the axillary recess, its reduction was a significant feature of frozen shoulder evaluated by MRA [6, 17]. However, there have been no reports concerning the relations between intra-articular structures and ROM restrictions by both MRI and MRA in the same patients with frozen shoulder.

The purpose of this study was to investigate correlations between the capsular changes and ROM restrictions evaluated by both plain MRI and MRA in the same patients with frozen shoulder. We hypothesized that MRA is useful in evaluating intra-articular structures by visualizing adhered scar tissues, and the axillary area in MRA and thickness of the capsule influenced ROM restrictions.

## 2 MATERIAL AND METHODS

### 2.1 Study Participants

The study protocols were approved by the institutional review board of the Iwate Prefectural Central Hospital (approval number: 2938). Between March 2015 and June 2016, 24 patients (13 male and 11 female patients) with frozen shoulder with severe ROM restriction who underwent both MRI and MRA preoperatively were included in this study. Their age ranged from 42 to 74 years (mean: 60.5 years, Standard Deviation [SD]: 8.6). Thickness of the CHL, thickness of the joint capsule in the axillary recess (humeral and glenoid portions), the maximal area of the axillary recess, and the capsular volume of the axillary recess were evaluated by both MRI and MRA. Passive ROM measurements, including FF, Lateral Elevation (LE), ER, 90° abduction with external rotation (AER), 90° Abduction with Internal Rotation (AIR), HBB, and horizontal flexion (HF) were measured in a standing position using a goniometer before MRI by a single orthopedic surgeon with 20 years of experience [20]. Hand behind the back was measured by asking the patients to place the thumb to the highest possible spinal vertebrae in the same position. We defined ROM restriction as FF < 120 ° and/or ER < 30° and/or HBB < L1 as previously reported [21, 22]. We excluded patients with rotator cuff tears, glenohumeral osteoarthritis, chronic joint arthritis, post-operation, diabetes mellitus, and/or previous fractures around the shoulder. Contrast agent leakage and unclear visualizations with MRA were also excluded.

### 2.2 MRI Arthrography Protocol

The patients underwent MRI with a 1.5-T imager (Signa HDxt 1.5T; GE Healthcare, Milwaukee, USA) with a phased-array surface coil. They were positioned with the humerus in a neutral position. T2-weighted MRI scans were obtained in axial, oblique coronal (perpendicular to the long axis of the glenohumeral joint), and oblique sagittal (parallel to the long axis of the glenohumeral joint) planes using a 3-mm slice thickness with a 1-mm gap between the slices. The MRI parameters of the T2 oblique coronal scans were as follows: Repetition Time (TR), 3020 ms; echo time (TE), 85 ms; Field Of View (FOV), 200 mm; and matrix, 256 · 256. For the T2 oblique sagittal scans, the parameters were TR, 3020 ms; TE, 85 ms; FOV, 200 mm; and matrix, 256 · 256.

**Fig. (1):**
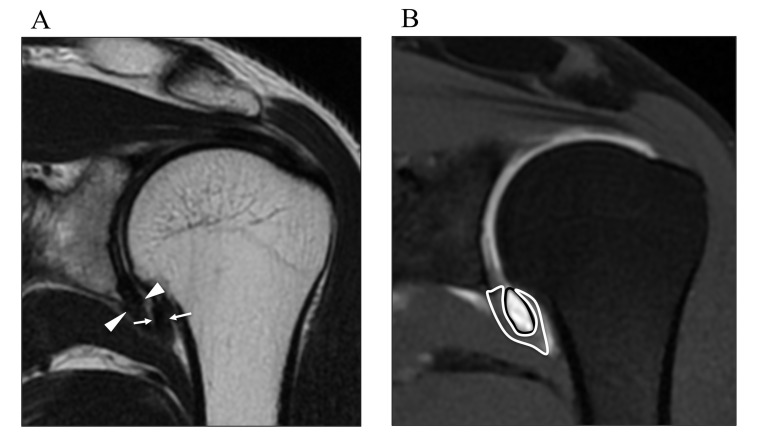
Measurements of thickness of the capsule and area of the axillary recess and capsule in the axillary recess.

Measurements were obtained on coronal oblique images, on both the thickest potion of capsule of humeral side (arrows) and glenoidal side (arrowheads) in the axillary recess (Fig. **1A**). The maximal area of the axillary recess (black line) and capsule (white line) in axillary recess is determined on coronal oblique images (Fig. **1B**).

Within 3 weeks after MRI scanning, an arthrogram procedure was performed, with a fluoroscopically guided shoulder joint injection of 10 ml of diluted gadolinium (Prohance gadoteridol, Bracco Diagnostics, Princeton, NJ, USA, diluted 1:100 with normal saline) into the shoulder joint. After the injection, MRA was performed with a phased array surface coil. Patients were positioned with the humerus in a neutral rotation. T1-weighted spin-echo images were obtained in the oblique coronal plane and sagittal oblique plane. The MRI parameters of the T1-weighted oblique coronal images were as follows: TR, 680 ms; TE, 9 ms; FOV, 200 mm; and matrix, 256 · 224. For the T1 oblique sagittal scans parameters were TR, 680 ms; TE, 9 ms; FOV, 200 mm; and matrix, 256 · 224.

### 2.3 Measurement of CHL

Orthopedic surgeons with 12 and 14 years of experience, who had no access to patients’ information, measured thickness of the CHL independently at its thickest point on the sagittal oblique T1-weighted spin-echo images as previously reported [5, 6]. MR images were analyzed with open-source Digital Imaging and Communications in Medicine software OsiriX MD (version 7.0, 64-bit). Measurements were taken to the nearest 0.1 mm, then rounded to the nearest millimeter. The average data of the two surgeons were used for the statistical analysis.

### 2.4 Measurement of Axillary Recess

As well as CHL, in the axillary recess, the thickest portion of both the humeral and glenoid sides were measured on coronal oblique images as previously reported [6] (Fig. **1A**). The maximal area of the axillary recess and the capsule at 6 o’clock in the axillary recess were determined on the coronal oblique images (Fig. **1B**).

### 2.5 Statistical Analysis

Capsular findings in axillary recess (*e.g*., thickness of the CHL, thickness of the capsule in the axillary recess, capsular area, and axillary area) between MRI and MRA were compared using the paired t-test. All continuous variables were tested for deviation from the normal distribution. The correlations between continuous variables without normal distributions were analyzed with the Spearman correlation coefficient and the correlations between variables with normal distributions were analyzed using the Pearson correlation coefficient. Data was expressed as mean ± SD (age, FF, LE, ER, AER, AIR, and HF) and medians and interquartile range (HBB). A value of p < 0.05 was accepted as statistically significant. All statistical analyses were performed with the statistical software package SPSS for Windows (version 23.0, SPSS Inc., Chicago, IL, USA). Values between 0.3 and 0.5 (-0.3 and -0.5) indicate a low correlation, between 0.5 and 0. (-0.5 and -0.7) indicate a moderate correlation, between 0.7 and 0.9 (-0.7 and -0.9) indicate a high correlation, and between 0.9 and 1.0 (−0.9 and −1.0) indicate a very high positive (negative) correlation.

## 3 RESULTS

Inter-observer reliability of thickness of the CHL (MRA; interclass correlation coefficients [ICC]: 0.895, 95% confidence interval (CI): 0.628–0.962, MRI; ICC: 0.864, 95% CI: 0.677–0.942), thickness of the capsule of glenoid side in axillary recess (MRA; ICC: 0.879, 95% CI: 0.722–0.948, MRI; ICC: 0.907, 95% CI: 0.8–0.959), thickness of the capsule of humeral side in axillary recess (MRA; ICC: 0.901, 95% CI: 0.786–0.956, MRI; ICC: 0.911, 95% CI: 0.805–0.960), capsular area (MRA; ICC: 0.916, 95% CI: 0.397–0.976, MRI; ICC: 0.931, 95% CI: 0.849–0.97), and the axillary area (MRA; ICC: 0.933, 95% CI: 0.853–0.971, MRI; ICC: 0.925, 95% CI: 0.828–0.968) were excellent.

The basic characteristics of the participants are shown in Table **1**. The average age of the females (64.4 years, SD: 5.2) was significantly higher compared with that of the males (57 years, SD: 10.3, p = 0.04). There was no significant difference in ROM between the male and female patients.

Results of the quantitative criteria and correlation coefficients between MRI and MRA imaging are shown in Table **2**. Thickness of the CHL was significantly higher in MRA than in MRI (p < 0.001). There is a low correlation in the thickness of the CHL (R = 0.38, p = 0.065). The capsular thickness of both the glenoid and humeral sides in the axillary recess significantly increased in MRI compared to MRA. (p = 0.009 and p < 0.001, respectively). The capsular area was significantly lower (p < 0.001) and the axillary area was significantly higher in MRA (p < 0.001) than in MRI. High correlations were observed in the capsular thickness of the axillary recess (glenoid side; R = 0.81, p<0.001, humeral side, R = 0.84, p < 0.001) and the capsular area (R = 0.84, p < 0.001), and a low correlation in the axillary area (R = 0.42, p = 0.044) between MRI and MRA (Table **2**).

The correlation coefficient between ROM and thickness of the CHL, the axillary area, capsular area, and thickness in the axillary recess on MRI and MRA are shown in Table **3**. A moderate negative correlation was found between thickness of the CHL and ER in MRA (R = -0.5, p = 0.014). Low positive correlations were found between the axillary area and FF (R = 0.43, p = 0.035) and ER (R = 0.43, p = 0.035) on MRA. Moderate correlations were found between the axillary area and LE (R = 0.66, p < 0.001), AER (R = 0.56, p = 0.004), and HBB (R = 0.6, p = 0.002) on MRA. Low negative correlations were found between the capsular area and HBB in both MRI (R = -0.42, p = 0.043) and MRA (R = -0.42, p = 0.044). Regarding thickness of the capsule in the axillary recess, negative correlations were found between the glenoid side and ER and HBB in both MRI and MRA (Table **3**). There were no significant correlations between ROM and thickness of the CHL and the axillary area in MRI, and no significant correlation between ROM and thickness of the humeral side capsule in the axillary recess in both MRI and MRA (Supplementary Table **1**).

## 4 DISCUSSION

The most important findings of this study were that most of the ROM restrictions were related to the axillary area in MRA, and thickness of the capsule at the glenoid side in the axillary recess was related to ER and HBB in both MRI and MRA. MRA also plays an important role in evaluating thickness of the CHL by visualizing adhered scar tissues when compared to MRI.

Frozen shoulder is a common disorder of shoulder characterized by pain and restricted shoulder motion in all planes. Its etiology is still unknown, but pathological changes in the joint capsule, such as fibrosis, inflammation, and chondrogenesis [23, 24], are one of the main factors for capsular changes, which are confirmed by an arthroscopic pancapsular release [25, 26].

Some reported that the RI and the surrounding structures, including the CHL, were involved in the pathogenesis of frozen shoulder [14, 27, 28]. The CHL attaches medially to the outer edge of the horizontal limb of the coracoid process and covers the RI [29], enveloping the entire SSP, ISP, and SSC muscles and their insertions [12]. The CHL consists of irregular and sparse fibers that have no boundary when compared to the superior glenohumeral ligament (SGHL), and contain abundant type III collagen, which provides flexibility to the ligament [12, 30]. The CHL has a significant effect on stability to the inferior and posterior translation of the humeral head and ROM in the shoulder from biomechanical studies [29, 31]. Thickening of the CHL with a dense fibrous tissue restricts ER [13, 14], which is in line with the finding of our study. Considering the restriction of ROM in internal rotation, the posterior capsular tightness was related to HBB in throwing athletes [32, 33] Further, thickening of the CHL or obliteration of subcoracoid fat triangles were correlated with FF, ER, and HBB in anterior glehohumeral instability [15]. However, there was no correlation between thickness of the CHL and HBB in this study. Thickening of the other parts of the joint capsule could change the humeral head position against the glenoid fossa and restricted ROM in frozen shoulder. A further study is needed to elucidate the positional relationship between the humeral head and glenoid fossa to ROM changes in frozen shoulder.

Characteristics of frozen shoulder are thickening of the joint capsule in the axillary recess and the CHL, and obliteration of the subcoracoid triangle evaluated by both MRI and MRA [5, 6, 10, 17, 18, 34]. However, little is known regarding the link between MRI findings and ROM restriction. The volume of articular fluid in MRI is not significantly diminished in frozen shoulder [18], along with the results of our study. As for the joint capsule in the axillary recess, more than 4 mm of the inferior glenohumeral ligament at the humeral side indicated adhesive capsulitis by MRI [18]. Recent reports showed that the joint capsule with edema at the humeral side of the axillary recess on fat-suppressed T2-weighted MR images was the most common finding in frozen shoulder, and this finding significantly correlated with ER restriction [34]. Significant negative correlations were observed between the thickness of the capsule at the glenoid side and ER, AER, and HBB in MRI. Not only the different methods of measurement and MRI conditions, but also the curved shape and an adhered point of the joint capsule could be easily changed by the position of the glenoid fossa and humeral head, which could explain the differences.

MRA can reveal decreased joint capacity of the frozen shoulder [35]. In this study, positive correlations were found between the axillary area and almost all ROMs. Thickening of the joint capsule in the axillary recess was correlated with ER restriction by MRA imaging [16]. However, other reports showed no correlations between thickness of the joint capsule and ROM restriction in frozen shoulder by MRA [5, 6, 19]. Negative correlations were observed between thickness of the capsule at the glenoid side and ER or HBB in MRA. Thickness of the capsule in the axillary recess, capsular area, and axillary area was thinner in MRA than in MRI, which could be explained by expansion of the joint capsule by the contrast agent. MRI showed a tendency to have a stronger correlation with the thickness of the joint capsule in the axillary recess when directly compared to MRA. The expansion of the joint capsule assumes to make an accurate evaluation of thickness and laxity of the joint capsule. HBB was related to the axillary area in MRA, capsular area in MRA and MRI, and thickness of the glenoid side capsule in MRA and MRI. HBB is an important factor for evaluating ROM in frozen shoulder. Given that HBB is an important motion in performing daily activities, such as lower leg dressing and washing and wiping backside, it is necessary to evaluate HBB restriction by physical examination and assuming its presence by MRI or MRA findings.

There are no reports comparing findings of MRI and MRA imaging in the same affected shoulder. Although MRA was useful for evaluating capsular changes from this study, it is an invasive and time-consuming method. However, it has a potential for joint distension, which can increase ROM [36]. The most notable determinant factor of the axillary pouch is the capsular area and the thickness of the glenoid side capsule by MRI. Regardless of injections of contrast medium, the axillary pouch is an important predictor for ROM restriction, which is a determinant factor for capsular release. Physical therapy or exercise intervention would not be effective for severe changes in the axillary pouch and CHL.

Limitations of present study are as follows: (1) no evaluation of arthroscopic findings, (2) no evaluation of the contralateral normal shoulder, (3) no data of the cases without surgery, and (4) comparison of limited MRI condition. Additionally, some MRI and MRA of patients could not be performed on the same day because of patient’s schedule. Further study is needed to clarify determinative factors for recalcitrant cases in frozen shoulder by MRI and MRA findings.

## CONCLUSION

The axillary area was significantly correlated with ROM restriction in FF, LE, ER, AER, and HBB in MRA. Thickness of the joint capsule at the glenoid side is an important factor for refractory frozen shoulder.

## Figures and Tables

**Table 1:** Basic population of characteristics (n=24).

	**N or Mean (SD)**
**Total**	24
**male**	13
**female**	11
**Age (years)**	60.5 (8.6)
**ROM (°)**	
**FF**	95 (19)
**LE**	92 (34)
**ER**	15 (27)
**AER**	60 (16)
**AIR**	-18 (40)
HBB^*^	L5 (L3-Buttock)
**HF**	97 (23)

FF Forward Flexion, LE Lateral Elevation, ER External Rotation, AER 90° Abduction with External Rotation, AIR 90° Abduction with Internal Rotation, HBB Hand Behind the Back, HF Horizontal Flexion, * Medians and interquartile ranges

**Table 2:** Quantitative criteria and correlation coefficient between MRI and MRA.

	MRI		MRA			Correlation Coefficient	
	Mean	SD	Mean	SD	P Value	R	P Value
**Thickness of CHL(mm)**	3.8	1.7	5.9	2.2	*<0.001*	0.38	0.065
**Thickness of capsule in axillary recess (mm)**							
**Glenoid side**	4.4	1.9	4.0	1.9	*0.009*	0.81	*<0.001*
**Humeral side**	4.4	1.7	3.7	1.8	*<0.001*	0.84	*<0.001*
**Capsular area (mm^2^)**	87.8	39.3	81.1	37.6	*<0.001*	0.84	*<0.001*
**Axillary area (mm^2^)**	10.3	9.7	30.4	23	*<0.001*	0.42	*0.044*

**Table 3:** Correlation coefficient difference between ROM and thickness of CHL, axillary area, capsular area and thickness of capsule in axillary recess.

			**FF**	**LE**	**ER**	**AER**	**AIR**	**HBB**	**HF**
**Thickness of CHL**	**MRA**	**R**	-0.22	-0.12	-0.5	-0.26	0.17	0.021	-0.023
		**P Value**	0.308	0.586	*0.014*	0.222	0.419	0.923	0.917
**Axillary area**	**MRA**	**R**	0.43	0.66	0.43	0.56	-0.22	0.6	0.07
		**P**	*0.035*	*< 0.001*	*0.035*	*0.004*	0.311	*0.002*	0.743
**Capsular Area**	**MRI**	**R**	-0.31	-0.12	-0.08	-0.13	0.01	-0.42	-0.08
		**P**	0.136	0.578	0.721	0.531	0.968	*0.043*	0.719
	**MRA**	**R**	−0.34	-0.15	-0.12	-0.19	−0.02	-0.42	-0.07
		**P**	0.104	0.49	0.567	0.384	0.911	*0.044*	0.761
**Thickness of capsule in axillary recess**									
**Glenoid side**	**MRI**	**R**	-0.35	-0.22	-0.59	-0.49	0.39	-0.44	0.06
		**P**	0.099	0.293	*0.002*	*0.016*	0.061	*0.034*	0.768
	**MRA**	**R**	-0.09	-0.11	-0.41	-0.3	0.25	-0.44	0.07
		**P**	0.676	0.601	*0.049*	0.159	0.241	*0.033*	0.743

## LIST OF ABBREVIATIONS

AER: = Abduction with External Rotation
AIR: = Abduction with Internal Rotation
CHL: = Coracohumeal Ligament
ER: = External Rotation
FF: = Delite
FF: = Forward Flexion
FOV: = Field of View
HBB: = Hand Behind the Back
HF: = Horizontal Flexion
IR: = Internal Rotation
ISP: = Intraspinatus
LE: = Lateral Elevation
MRA: = Magnetic Resonance Arthrography
MRI: = Magnetic Resonance Imaging
RI: = Rotator Interval
ROM: = Range of Motion
SD: = Standard Deviation
SGHL: = Superior Glenohumeral Ligament
SSC: = Subscapularis
SSP: = Supraspinatus
TE: = Echo Time
TR: = Repetition Time

## References

[1] Zuckerman J.D., Rokito A. (2011). Frozen shoulder: A consensus definition.. J. Shoulder Elbow Surg..

[2] Andersen N.H., Søjbjerg J.O., Johannsen H.V., Sneppen O. (1998). Frozen shoulder: Arthroscopy and manipulation under general anesthesia and early passive motion.. J. Shoulder Elbow Surg..

[3] Pollock R.G., Duralde X.A., Flatow E.L., Bigliani L.U. (1994). The use of arthroscopy in the treatment of resistant frozen shoulder.. Clin. Orthop. Relat. Res..

[4] Yoo J.C., Ahn J.H., Lee Y.S., Koh K.H. (2009). Magnetic resonance arthrographic findings of presumed stage-2 adhesive capsulitis: Focus on combined rotator cuff pathology.. Orthopedics.

[5] Lee S.Y., Park J., Song S.W. (2012). Correlation of MR arthrographic findings and range of shoulder motions in patients with frozen shoulder.. AJR Am. J. Roentgenol..

[6] Mengiardi B., Pfirrmann C.W., Gerber C., Hodler J., Zanetti M. (2004). Frozen shoulder: MR arthrographic findings.. Radiology.

[7] Chung C.B., Dwek J.R., Cho G.J., Lektrakul N., Trudell D., Resnick D. (2000). Rotator cuff interval: Evaluation with MR imaging and MR arthrography of the shoulder in 32 cadavers.. J. Comput. Assist. Tomogr..

[8] Morag Y., Jacobson J.A., Shields G., Rajani R., Jamadar D.A., Miller B., Hayes C.W. (2005). MR arthrography of rotator interval, long head of the biceps brachii, and biceps pulley of the shoulder.. Radiology.

[9] Iannotti J.P. (1994). Evaluation of the painful shoulder.. J. Hand Ther..

[10] Li J.Q., Tang K.L., Wang J., Li Q.Y., Xu H.T., Yang H.F., Tan L.W., Liu K.J., Zhang S.X. (2011). MRI findings for frozen shoulder evaluation: Is the thickness of the coracohumeral ligament a valuable diagnostic tool?. PLoS One.

[11] Zhao W., Zheng X., Liu Y., Yang W., Amirbekian V., Diaz L.E., Huang X. (2012). An MRI study of symptomatic adhesive capsulitis.. PLoS One.

[12] Arai R., Nimura A., Yamaguchi K., Yoshimura H., Sugaya H., Saji T., Matsuda S., Akita K. (2014). The anatomy of the coracohumeral ligament and its relation to the subscapularis muscle.. J. Shoulder Elbow Surg..

[13] Neer C.S., Satterlee C.C., Dalsey R.M., Flatow E.L. (1992). The anatomy and potential effects of contracture of the coracohumeral ligament.. Clin. Orthop. Relat. Res..

[14] Ozaki J., Nakagawa Y., Sakurai G., Tamai S. (1989). Recalcitrant chronic adhesive capsulitis of the shoulder. Role of contracture of the coracohumeral ligament and rotator interval in pathogenesis and treatment.. J. Bone Joint Surg. Am..

[15] Kanazawa K., Hagiwara Y., Kawai N. (2016). Correlations of coracohumeral ligament and range of motion restriction in patients with recurrent anterior glenohumeral instability evaluated by magnetic resonance arthrography.. J. Shoulder Elbow Surg..

[16] Ahn K.S., Kang C.H., Oh Y.W., Jeong W.K. (2012). Correlation between magnetic resonance imaging and clinical impairment in patients with adhesive capsulitis.. Skeletal Radiol..

[17] Jung J.Y., Jee W.H., Chun H.J., Kim Y.S., Chung Y.G., Kim J.M. (2006). Adhesive capsulitis of the shoulder: Evaluation with MR arthrography.. Eur. Radiol..

[18] Emig E.W., Schweitzer M.E., Karasick D., Lubowitz J. (1995). Adhesive capsulitis of the shoulder: MR diagnosis.. AJR Am. J. Roentgenol..

[19] Yoon J.P., Chung S.W., Lee B.J. (2015). Correlations of magnetic resonance imaging findings with clinical symptom severity and prognosis of frozen shoulder.. Knee Surg. Sports Traumatol. Arthrosc..

[20] Hagiwara Y., Ando A., Kanazawa K., Koide M., Sekiguchi T., Hamada J., Itoi E. (2017). Arthroscopic coracohumeral ligament release for patients with frozen shoulder.. Arthrosc. Tech..

[21] Audigé L., Blum R., Müller A.M., Flury M., Durchholz H. (2015). Complications following arthroscopic rotator cuff tear repair: A systematic review of terms and definitions with focus on shoulder stiffness.. Orthop. J. Sports Med..

[22] Oh J.H., Kim S.H., Lee H.K., Jo K.H., Bin S.W., Gong H.S. (2008). Moderate preoperative shoulder stiffness does not alter the clinical outcome of rotator cuff repair with arthroscopic release and manipulation.. Arthroscopy.

[23] Hagiwara Y., Ando A., Onoda Y., Takemura T., Minowa T., Hanagata N., Tsuchiya M., Watanabe T., Chimoto E., Suda H., Takahashi N., Sugaya H., Saijo Y., Itoi E. (2012). Coexistence of fibrotic and chondrogenic process in the capsule of idiopathic frozen shoulders.. Osteoarthritis Cartilage.

[24] Lundberg B.J. (1970). Glycosaminoglycans of the normal and frozen shoulder-joint capsule.. Clin. Orthop. Relat. Res..

[25] Harryman D.T. (1993). Shoulders: Frozen and stiff.. Instr. Course Lect..

[26] Warner J.J., Allen A., Marks P.H., Wong P. (1996). Arthroscopic release for chronic, refractory adhesive capsulitis of the shoulder.. J. Bone Joint Surg. Am..

[27] Hand G.C., Athanasou N.A., Matthews T., Carr A.J. (2007). The pathology of frozen shoulder.. J. Bone Joint Surg. Br..

[28] Neviaser J.S. (1962). Adhesive capsulitis of the shoulder (the frozen shoulder).. Med. Times.

[29] Harryman D.T., Sidles J.A., Harris S.L., Matsen F.A. (1992). The role of the rotator interval capsule in passive motion and stability of the shoulder.. J. Bone Joint Surg. Am..

[30] Arai R., Mochizuki T., Yamaguchi K., Sugaya H., Kobayashi M., Nakamura T., Akita K. (2010). Functional anatomy of the superior glenohumeral and coracohumeral ligaments and the subscapularis tendon in view of stabilization of the long head of the biceps tendon.. J. Shoulder Elbow Surg..

[31] Boardman N.D., Debski R.E., Warner J.J., Taskiran E., Maddox L., Imhoff A.B., Fu F.H., Woo S.L. (1996). Tensile properties of the superior glenohumeral and coracohumeral ligaments.. J. Shoulder Elbow Surg..

[32] Burkhart S.S., Morgan C.D., Kibler W.B. (2003). The disabled throwing shoulder: Spectrum of pathology Part I: Pathoanatomy and biomechanics.. Arthroscopy.

[33] Tehranzadeh A.D., Fronek J., Resnick D. (2007). Posterior capsular fibrosis in professional baseball pitchers: Case series of MR arthrographic findings in six patients with glenohumeral internal rotational deficit.. Clin. Imaging.

[34] Park S., Lee D.H., Yoon S.H., Lee H.Y., Kwack K.S. (2016). Evaluation of adhesive capsulitis of the shoulder with fat-suppressed T2-weighted MRI: Association between clinical features and MRI findings.. AJR Am. J. Roentgenol..

[35] Neviaser J.S. (1962). Arthrography of the shoulder joint: Study of the findings in adhesive capsulitis of the shoulder. Study of the findings in adhesive capsulitis of the shoulder.. J. Bone Joint Surg. Am..

[36] Park Y.H., Park Y.S., Chang H.J., Kim Y. (2016). Correlations between MRI findings and outcome of capsular distension in adhesive capsulitis of the shoulder.. J. Phys. Ther. Sci..

