# Supplementary material for: Correlations between Capsular Changes and ROM Restriction in Frozen Shoulder Evaluated by Plain MRI and MR Arthrography
Source: Open Orthop J. 2018 Oct 17;12:396–404. doi: 10.2174/1874325001812010396 (PMC6198418; doi:10.2174/1874325001812010396)
Supplement: Supplementary file 1 [file TOORTHJ-12-396_SD1.pdf]

Send Orders for Reprints to reprints@benthamscience.ae

BENTHAM OPEN

CrossMark

The Open Orthopaedics Journal

Content list available at: www.benthamopen.com/TOORTHJ/

DOI: 10.2174/1874325001812010396, 2018, *12*, 03-00

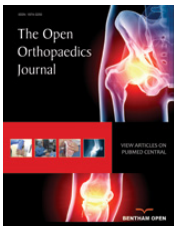

RESEARCH ARTICLE

Correlations between Capsular Changes and ROM Restriction in Frozen Shoulder Evaluated by Plain MRI and MR Arthrography

Kenji Kanazawa[1], Yoshihiro Hagiwara[2,*], Takuya Sekiguchi[3], Kazuaki Suzuki[4], Masashi Koide[5], Akira Ando[5] and Yutaka Yabe[2]

[1]*Department of Orthopaedic Surgery, South Miyagi Medical Center, Oogawara, Miyagi, Japan*
[2]*Department of Orthopaedic Surgery, Tohoku University School of Medicine, Sendai, Miyagi, Japan*
[3]*Department of Orthopaedic Surgery, Iwate Prefectural Central Hospital, Morioka, Iwate, Japan*
[4]*Department of Orthopaedic Surgery, JR Sendai Hospital, Sendai, Miyagi, Japan*
[5]*Department of Orthopaedic Surgery, Matsuda Hospital, Sendai, Miyagi, Japan*

Received: June 8, 2018 Revised: September 13, 2018 Accepted: September 13, 2018

Supplementary Table 1. Correlation coefficient difference between ROM and thickness of CHL, axillary area, capsular area and thickness of capsule in axillary recess.

			FF	LE	ER	AER	AIR	HBB	HF
**Thickness of CHL**	**MRI**	**R**	-0.31	-0.08	-0.24	0.09	0.28	-0.1	0.1
		**P**	0.139	0.714	0.269	0.681	0.18	0.639	0.65
**Axillary area**	**MRI**	**R**	0.19	0.36	0.25	0.24	-0.21	0.18	0.06
		**P**	0.385	0.087	0.235	0.255	0.337	0.4	0.768
**Thickness of capsule in axillary recess**									
**Humeral side**	**MRI**	**R**	0.13	<0.01	-0.2	-0.11	0.09	-0.1	0.12
		**P**	0.559	0.995	0.339	0.607	0.673	0.625	0.57
	**MRA**	**R**	0.01	<-0.01	-0.08	0.01	−0.02	<0.01	0.03
		**P**	0.954	0.999	0.702	0.973	0.942	0.986	0.872

* Address correspondence to this author at the Department of Orthopaedic Surgery, Tohoku University School of Medicine, 1-1 Seiryo-machi, Aoba-ku, Sendai, 980-8574, Japan; Tel: +81-22-717-7245; Fax: +81-22-717-7248; E-mail: hagi@med.tohoku.ac.jp

© 2018 Kanazawa *et al*.

This is an open access article distributed under the terms of the Creative Commons Attribution 4.0 International Public License (CC-BY 4.0), a copy of which is available at: (https://creativecommons.org/licenses/by/4.0/legalcode). This license permits unrestricted use, distribution, and reproduction in any medium, provided the original author and source are credited.
